# The Impact of Psyching-Up and Cognitive Challenges on Cognitive Performance and Well-Being in Adolescent Swimmers: A Randomized Controlled Trial

**DOI:** 10.3390/children12121591

**Published:** 2025-11-23

**Authors:** Yasmine Dhaouadi, Riadh Khalifa, Antonella Muscella

**Affiliations:** 1 Higher Institute of Sport and Physical Education of Ksar Said, University of La Manouba, Tunis 2010, Tunisia; yassmin.dhaouadi1@gmail.com (Y.D.); riadhkhal@yahoo.fr (R.K.); 2 Department of Biological and Environmental Science and Technologies (DiSTeBA), University of Salento, 73100 Lecce, Italy

**Keywords:** psyching-up techniques, cognitive training, adolescent swimmers, cognitive performance, psychological well-being

## Abstract

**Highlights:**

**What are the main findings?**

- Integrating psyching-up and cognitive challenges in swimming training improved cognitive performance, mood regulation, and exercise enjoyment in adolescent swimmers.
- The Psychological Cognitive Group (PCG) exhibited enhanced reaction time, accuracy, and impulse control, with no additional physiological strain compared to other groups.

**What is the implication of the main finding?**

- Combining psychological and cognitive interventions can optimize both athletic performance and psychological well-being, offering a potential framework for enhancing training programs.
- The findings support the importance of psychological skills training in youth sports, suggesting that it can be used to improve mental resilience and enjoyment without increasing physical load.

**Abstract:**

Background/Objectives: The integration of psychological techniques, such as psyching-up, into sports training has been increasingly explored for its potential to enhance athletic performance and cognitive function, especially in young athletes. This study aimed to examine the effects of combining psyching-up techniques with cognitive challenges on psychophysiological responses and visuo-auditory attention in adolescent competitive swimmers. Methods: A total of 48 male competitive swimmers were randomly assigned to three groups: the Psyching-Up and Cognitive Group (PCG), the Cognitive Training Group (CGT), and a Control Group (CG). The intervention involved ten training sessions, where the PCG received psyching-up techniques, while both the PCG and CGT participated in cognitive training tasks. Key assessments included cognitive performance tests (Bells Test, Trail Making Test Parts A and B, Go/No-Go Auditory Task), heart rate (%HR max), blood lactate levels, perceived exertion (RPE), and mood state (Total Mood Disturbance). Results: The PCG showed significant improvements in cognitive performance, with fewer omissions in the Bells Test (*p* = 0.041) and faster reaction times in the Trail Making Test (Part A, *p* = 0.002; Part B, *p* = 0.001). In the Go/No-Go Auditory Task, the PCG exhibited faster reaction times and a higher hit rate (*p* = 0.001). There were no significant differences in physiological responses, with %HR max and blood lactate levels showing stable trends across groups. However, the PCG reported significantly higher enjoyment (*p* < 0.001) and a reduction in Total Mood Disturbance (*p* < 0.001). Conclusions: Integrating psyching-up techniques with cognitive challenges positively impacts cognitive performance and psychological well-being in adolescent swimmers, without altering physiological responses. These findings highlight the potential of psychological interventions to enhance performance and overall athlete experience in youth sports training.

## 1. Introduction

Competitive swimming performance requires the integration of physiological capacity, biomechanical efficiency, and cognitive processing [1,2]. Optimal performance is contingent upon the effective regulation of physiological arousal, sustained attentional focus under conditions involving concurrent sensory stimuli, and precise execution of motor skills during physiological stress [3,4]. Adolescence is characterized by significant neuromaturational development, encompassing physiological growth, cognitive maturation, and emotional regulation processes [5,6]. Adolescent swimmers concurrently face increasing competitive demands, ongoing physiological adaptations, and developmental changes in attentional control systems [7,8,9]. Conventional swimming training methodologies predominantly emphasize physiological adaptation through overload, potentially neglecting the systematic optimization of psychophysiological states and the development of cognitive resilience under sport-specific, high-demand conditions [3,10,11].

Existing evidence indicates that a well-developed sport-specific training background is strongly associated not only with enhanced physical performance but also with superior postural control, sensorimotor integration, and cognitive resilience. Athletes with advanced motor skills, proprioceptive awareness, and core stability demonstrate greater adaptability and consistency when performing under high-pressure or fatiguing conditions, highlighting a dynamic interdependence between sensorimotor coordination, postural stability, and executive functions such as attentional control, decision-making, and working memory [12,13,14]. Moreover, training programs that deliberately incorporate balance, core stabilization, and proprioceptive exercises have been shown to improve postural alignment, neuromuscular efficiency, and dual-task performance, which in turn enhances the capacity to maintain focus, process complex stimuli, and make rapid sport-specific decisions during competition [12,15]. These findings collectively underscore that structured interventions targeting both physical and cognitive domains can reinforce the link between physical preparedness and cognitive resilience, providing athletes with a more robust foundation for coping with the multifaceted demands of sport-specific performance.

Within this context, psyching-up (PU) techniques, defined as deliberate self-regulation strategies including motivational self-talk, imagery, breathing control, and preparatory arousal, constitute empirically supported methods for athletes to modulate arousal states, self-efficacy, and attentional focus, thereby enhancing preparatory states and performance outcomes. Concurrently, introducing cognitive challenges (CCs) during physical training, designed to simulate competition-specific attentional demands (e.g., executing decision-making tasks under physiological fatigue, processing task-relevant external stimuli), provides a method for enhancing cognitive resilience and dual-task performance efficiency [16,17,18,19,20,21]. Recent meta-analyses and reviews emphasize the effectiveness of psychological skills training in young athletes, highlighting improvements in attentional control, decision-making, and stress regulation [22,23].

While empirical evidence supports the efficacy of isolated PU strategies and CC paradigms across various athletic domains [23,24,25,26], the systematic integration of these approaches within aquatic training environments, characterized by high-sensory load and specifically applied to adolescent populations, has received limited empirical investigation. This lack of integrated research represents a gap in understanding whether the concurrent application of PU and CC elicits additive or interactive effects on performance readiness, potentially offering a more comprehensive preparation model for the concurrent physiological, cognitive, and environmental demands of competition.

Crucially, the effectiveness of such integrated interventions must be evaluated through important psychophysiological and cognitive lenses.

Psychophysiological responses (e.g., perceived exertion (RPE) and affective valence) provide objective and subjective markers of stress, arousal regulation, and effort [27,28]. Simultaneously, visuo-auditory attention, defined as the capacity to selectively process and respond to relevant visual and auditory stimuli while filtering distractions [29,30], is paramount in the aquatic environment. Understanding how integrated PU and CC training influences these interconnected systems is vital for developing truly holistic training protocols.

Therefore, this study investigated the novel integration of psyching-up techniques and cognitive challenges within the regular swimming training regimen of adolescent athletes. We specifically examined the synergistic effects of this combined intervention on key psychophysiological responses (including stress markers, autonomic regulation, and perceived states) and visuo-auditory attentional performance, compared to standard swimming training or training incorporating only one component (PU or CC alone). We hypothesize that the integrated PU and CC approach will elicit more favorable psycho-physiological adaptations, indicative of improved arousal regulation and stress resilience under training load. Additionally, we expect it to significantly enhance visuo-auditory attentional performance during and after exercise compared to the control and single-component conditions.

## 2. Materials and Methods

### 2.1. Participants

Firstly, 54 adolescent competitive swimmers were recruited from three regional swimming clubs. Participants were recruited through direct collaboration with club coaches, who distributed information letters and consent forms to athletes and their parents. All volunteers who met the inclusion criteria and provided written informed consent were considered for enrollment.

Inclusion criteria were: (1) age between 12 and 14 years; (2) at least one year of competitive swimming experience (minimum of three sessions per week); (3) physical and cognitive ability to complete all procedures, and (4) absence of neurological, sensory, or musculoskeletal disorders. Exclusion criteria included recent injuries, unmanaged psychological or attention disorders, use of psychoactive medication, or participation in other structured cognitive programs.

Based on the described selection criteria, a total of 48 adolescent swimmers (mean age: 13.02 ± 0.76 years; height: 157.4 ± 7.8 cm; body mass: 48.3 ± 6.9 kg; body fat percentage: 14.7 ± 2.3%) were included in the study. Only male participants were enrolled to control for potential confounding effects of sex-based hormonal fluctuations during adolescence, which could influence mood, stress reactivity, and cognitive performance.

The sample size determination was performed using software G*Power version 3.1.9.7 (Heinrich-Heine-Universität Düsseldorf, Germany), targeting a medium effect size (f = 0.23), alpha level of (α = 0.05), and power of (1 − β) = 0.86 for a repeated-measures ANOVA with between- and within-subject factors. The analysis indicated a minimum required sample of 42 participants; thus, 48 were recruited to ensure adequate statistical power and account for potential dropouts.

Randomization was carried out using Research Randomizer (www.randomizer.org, accessed on 2 March 2025).

**Figure 1:**
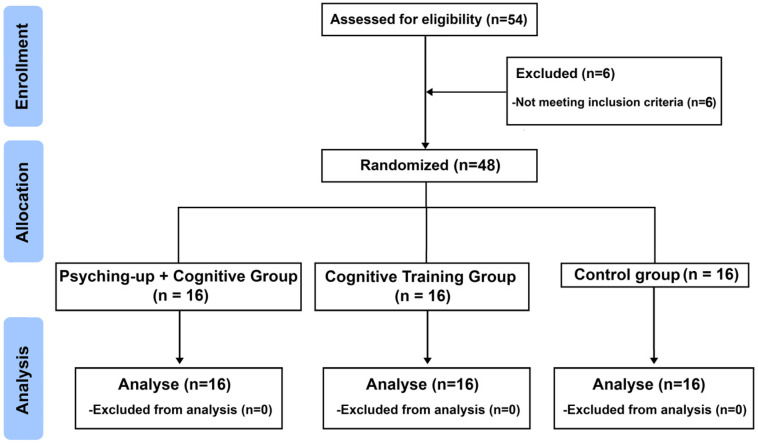
Flowchart of study.

A stratified permuted block design was applied, with stratification based on age and competitive swimming experience, in order to control for differences in training background and developmental stage. Within each stratum, participants were randomly assigned in blocks of six to one of the three groups: the Psyching-up and Cognitive Group (PCG, *n* = 16), which received both mental preparation through psyching-up techniques and cognitive task-integrated swimming training; the Cognitive Training Group (CGT, *n* = 16), which performed the same swimming tasks without the psyching-up component; and the Control Group (CG, *n* = 16), which engaged in traditional swimming training without any psychological or cognitive additions (Figure 1). Allocation concealment was maintained by an independent researcher who was not involved in participant recruitment or data collection.

Before participation, written informed consent was obtained from parents or legal guardians, and verbal assent was obtained from all swimmers, in accordance with the Declaration of Helsinki and local ethical regulations. The study protocol was approved by the Institutional Review Board (ISSEPK-009/2025). Attendance and participation rates were recorded for all participants, with attendance above 95% in all groups. Reasons for any missed sessions (e.g., minor illness) were documented. No participants withdrew from the study; therefore, drop-out analysis was not applicable.

Due to the nature of the intervention, participants and coaches were aware of group allocation. However, all outcome assessors and data analysts were blinded to group assignment. Cognitive and performance assessments were conducted by researchers not involved in training delivery, and data were coded before analysis to ensure unbiased evaluation.

### 2.2. Experimental Protocol

This study is a three-arm, parallel-group randomized controlled trial conducted in accordance with the CONSORT guidelines. The PCG group (Psyching-up and Cognitive Group) participated in a comprehensive intervention that integrated both psyching-up techniques (e.g., self-talk, visualization, motivational routines) and embedded cognitive tasks. In contrast, the CGT group (Cognitive Task Group) engaged in the same swimming exercises with embedded cognitive tasks but without the psyching-up preparation. In addition, the Control Group (CG) followed a traditional swimming training program consisting of standard stroke drills, endurance work, and technique development, with no cognitive or mental training components. All groups were trained under identical environmental conditions (same pool, time of day, instructors, water, and air temperature).

### 2.3. Program Training

**Figure 2:**
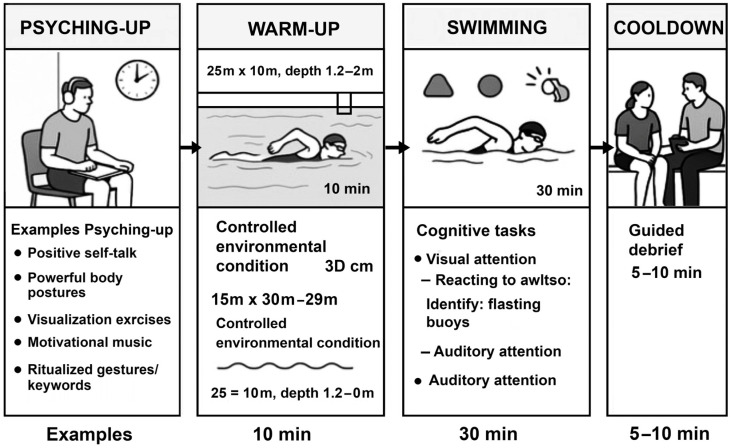
Integrated Swimming Session with Psyching-Up and Cognitive Tasks.

The program involved 10 sessions of training; each session was conducted in a standard 25 m by 10 m swimming pool, with a depth ranging from 1.2 to 2.0 m. Environmental conditions were carefully controlled, maintaining water temperatures between 27 °C and 29 °C and ambient air temperatures between 24 °C and 26 °C to ensure comfort and physiological safety. The sessions were scheduled twice per week over five weeks, with each session lasting approximately 60 min. A critical component of the program is the 30 min pre-session psyching-up phase, which takes place in a calm, quiet designated mental preparation room near the pool, supervised by a sports psychologist and mental trainer. During this phase, athletes are guided through evidence-based psyching-up techniques aimed at enhancing arousal, focus, motivation, and emotional readiness. These techniques vary across sessions and include positive self-talk (e.g., repeating phrases like “I am ready” or “I am focused”), powerful body postures combined with fast, rhythmic breathing, dynamic visualization exercises where the swimmer mentally rehearses success scenarios, listening to motivational music via headphones or speakers, and ritualized gestures or keywords (e.g., shouting “Go!” or clapping) used to create a consistent performance mindset. Other techniques include peer-to-peer encouragement rituals, motivational speeches by the coach, and silent mental rehearsal of the task ahead. After this mental activation, swimmers proceed to the pool where they begin with a 10 min warm-up involving light swimming across various strokes (freestyle, backstroke, breaststroke) to prepare the cardiovascular and muscular systems. The core of each session is a 30 min swimming block where cognitive tasks are embedded directly into aquatic drills, targeting specific attentional systems. These tasks are carefully selected and gradually intensified to suit the cognitive and physical levels of the swimmers. For visual attention, tasks include identifying the color of floating buoys, reacting to flashing lights on the poolside, recognizing underwater hand signals, or spotting symbols posted along the walls mid-lap. For auditory attention, tasks involve reacting to whistle tones of different pitches (e.g., high tone = continue, low tone = stop), following rhythmic clapping patterns, or responding to verbal instructions with immediate motor adaptation. Some tasks demand dual-task processing, where swimmers must, for example, adjust their swimming rhythm based on changing auditory cues or remember visual sequences presented just before a dive. Cognitive inhibition and working memory are also targeted through stop-and-go commands or multi-step auditory directions delivered during laps. Each session concludes with a 5 to 10 min cooldown phase and a guided debrief, during which athletes reflect on their performance, describe their attentional experience, and report on subjective mental load, motivation, and enjoyment. This feedback is used by the coaching staff to adjust the intensity and complexity of subsequent sessions (Figure 2).

The first session begins with positive self-talk, such as repeating “I am focused” before performing freestyle intervals. During the swim, swimmers are required to identify the color of floating cones, train their visual scanning, and pay selective attention. In the second session, the athlete engages in fast breathing combined with a power pose (e.g., arms raised in a victory stance) before performing backstroke kicking drills. The cognitive challenge includes responding to flashing lights on the pool’s edge, enhancing visual reactivity. The third session introduces motivational music during warm-up and team claps to build energy, followed by breaststroke laps where the swimmer listens for the coach to call out a color and responds by clapping once or twice, targeting auditory discrimination and memory.

In session four, the psyching-up method involves positive visualization, where the swimmer imagines a successful underwater glide. During the task, the coach shows fingers under the water, and the swimmer must accurately count them, stimulating visual concentration and near-vision accuracy. Session five uses a ritualized scream and jump, a common psyching-up method in team sports, to enhance emotional readiness before a relay race. During the swim, swimmers respond to whistle sounds with different pitches, requiring auditory cue recognition and motor inhibition. Session six leverages an energetic 3-2-1 countdown as a psyching-up tool, paired with dive start practice. Swimmers must also memorize and recall a sequence of visual cards shown just before the dive, engaging visual working memory.

Session seven focuses on peer encouragement, where swimmers motivate one another before starting an obstacle swim course. This session includes sudden auditory cues (e.g., buzzer sounds) that prompt the swimmer to change direction or stop, enhancing auditory attention and reaction speed. Session eight emphasizes the silent repetition of motivational scripts during synchronized swimming. Here, the swimmer adjusts stroke timing in response to the coach altering the rhythm, a task requiring auditory attention and rhythmic adaptation. In session nine, the coach delivers a motivational speech before a timed lap challenge, during which swimmers must briefly identify a shape drawn on a wall—a task that strengthens rapid visual detection and shape recognition under physical stress.

Finally, session ten includes mental rehearsal, where swimmers mentally simulate their entire swim performance before engaging in a mixed stroke challenge. During this exercise, they follow multi-step verbal commands provided by the coach, engaging both auditory and visual attention in a dual task setting. This final session integrates all previously developed skills under complex and realistic conditions, reinforcing the transfer of cognitive and attentional gains to real competition scenarios (Table 1).

### 2.4. Data Collection

A comprehensive and multidimensional data collection protocol was implemented to assess the cognitive, physiological, and psychological responses of participants throughout the intervention.

Measurements were performed in a controlled swimming pool for physical performance tests and in a quiet room for cognitive assessments. Assessments were conducted at three time points: before the first session (pre-test, T1), after the fifth session (mid-test, T2), and after the final session (post-test, T3).

All data collection and training sessions took place during the same period of the competitive swimming season (April–May) over five consecutive weeks. Sessions were scheduled at the same time of day, between 4:00 p.m. and 6:00 p.m., corresponding to the athletes’ regular training schedule, to minimize variability due to circadian factors and fatigue. Environmental conditions were standardized for all participants, with water temperature maintained between 27–29 °C and air temperature between 24–26 °C.

Data collection was performed by trained researchers specializing in sport and exercise psychology, under the supervision of the principal investigator, while physiological measures (heart rate, lactate) were collected and analyzed by certified sports scientists. Importantly, all data analysts were blinded to group allocation to minimize bias during data processing and statistical analyses.

Cognitive performance was evaluated through standardized and validated neuropsychological tests. Visual attention was assessed using the Bells Test (measuring selective visual scanning and processing speed) and the Trail Making Test parts A and B (evaluating visual-motor tracking and attention shifting), while auditory attention was examined through a computerized Go/No-Go auditory discrimination task, requiring rapid response inhibition and discrimination of variable pitch sounds. This task also provided measures of auditory working memory and sequencing ability.

To monitor exercise intensity, heart rate (%HRmax) was continuously recorded during all swimming sessions using Polar H10 chest strap monitors. Data were expressed as a percentage of each participant’s estimated maximal heart rate (%HRmax), calculated using the Tanaka formula: 208 − (0.7 × age). Mean and peak %HRmax values were analyzed to compare training load across sessions and between groups. Additionally, blood lactate concentration was measured using the Lactate Pro 2 portable analyzer. Capillary blood samples (0.5 µL) were collected from the earlobe 3 min after the last swimming bout of each session to capture acute lactate accumulation and infer anaerobic effort.

Perceived exertion was measured 15 min after the last swimming bout of each session using the Borg CR-10 Scale. Furthermore, physical activity enjoyment was evaluated 20 min after each session using the Physical Activity Enjoyment Scale (PACES).

To capture emotional fluctuations and psychological responses to training, mood state was assessed using the Brunel Mood State Scale (BRUMS), adapted for pre- and post-session administration. Participants completed the BRUMS immediately before and after each training session, indicating how they felt in terms of happiness, tension, tiredness, and calmness. This assessment provided insight into how different training modalities influenced affective states over the course of the intervention.

Cognitive and psychological instruments were administered in the participants’ native Arabic language. An Arabic version of the BRUMS has previously been validated among Tunisian athletes [31]. For the PACES, no specific Tunisian Arabic version is available; we used a linguistically translated version following forward-backward translation procedures and pilot-tested it in our sample for clarity and comprehension [32].The Bells Test, TMT-A/B, and the Go/No-Go auditory task were administered in their standard format; instructions and stimuli were translated and checked for comprehension by Tunisian researchers fluent in Arabic. The Borg CR-10 scale was applied following its universal format, which has been validated across different cultural contexts. All instructions were provided by trained researchers fluent in the participants’ language to ensure full understanding and consistency.

### 2.5. Cognitive Measurements

#### 2.5.1. Bells Test

The Bells Test was used to assess visual attention and detect potential signs of visual neglect in adolescent swimmers participating in the study [33]. This standardized neuropsychological tool evaluates an individual’s ability to scan visually and selectively attend to specific stimuli within a structured visual array.

The test consists of an A4 sheet containing 315 small figures, including 35 bell symbols randomly distributed among various distractors (e.g., houses, apples, horses). Participants were instructed to circle all the bells they could find as quickly and accurately as possible within a 120 s time limit. The sheet was divided into seven vertical columns, allowing for an assessment of visual scanning patterns from left to right.

Before administration, standardized instructions were provided to ensure full task comprehension. During testing, participants were asked to start from the top left corner and proceed systematically to the bottom right of the page, identifying and circling each bell.

Scoring included the total number of bells correctly identified (maximum score = 35), the number of omissions (bells not circled), and completion time in seconds. The omission score serves as the primary indicator of attentional deficits, while lateralized omission patterns may suggest hemispatial neglect. A higher number of correctly identified bells indicates better visual selective attention and concentration.

#### 2.5.2. TMT—Part A

The Trail Making Test Part A (TMT-A) was employed to assess processing speed, visual scanning, attention, and psychomotor efficiency in adolescent swimmers [34]. This standardized neuropsychological test is widely recognized for evaluating cognitive flexibility and attention-related functions in both youth and adult populations.

In Part A, participants were presented with a sheet containing 25 numbered circles (1–25) randomly distributed across the page. They were instructed to draw a continuous line connecting the numbers in ascending order (i.e., 1–2–3…25) as quickly and accurately as possible, without lifting the pen from the paper. Standardized instructions were provided, and a brief practice trial was administered to ensure task comprehension.

Timing began when the participant started connecting the first two numbers and stopped when circle 25 was reached. The primary outcome measure is the total completion time, expressed in seconds. Errors, such as connecting an incorrect sequence (e.g., skipping a number), were immediately corrected by the examiner during administration but recorded for qualitative analysis. Shorter completion times indicate greater visual attention, faster processing speed, and improved psychomotor coordination.

#### 2.5.3. TMT Test Part B

The Trail Making Test Part B (TMT-B) was employed to assess executive functioning, particularly cognitive flexibility, task-switching ability, and divided attention in adolescent swimmers [34]. This widely used neuropsychological test challenges participants to alternate between two cognitive sets under time constraints.

The test sheet included 13 numbered circles (1–13) and 12 letters (A–L) randomly distributed across an A4 page. Participants were instructed to connect the circles in an alternating numeric–alphabetic sequence (i.e., 1–A–2–B–3–C…13–L) by drawing a continuous line as quickly and accurately as possible, without lifting the pen. This alternation engages working memory, attentional control, and mental flexibility.

Before testing, standardized instructions were provided, and a brief practice trial (e.g., 1–A–2–B–3–C) was administered to ensure task comprehension. Completion time (in seconds) was recorded using a stopwatch, starting when the participant began the sequence and ending upon reaching the final target (L). Errors—such as incorrect sequencing or skipped items—were immediately corrected by the examiner, while the number of mistakes was documented for qualitative analysis.

The primary outcome measure is total completion time, with shorter times indicating better executive functioning. Conversely, longer completion times or frequent sequencing errors may reflect reduced cognitive flexibility, divided attention, or inhibitory control.

#### 2.5.4. Go/No-Go Auditory Task

The Go/No-Go Auditory Task (computerized version) was employed to assess auditory attention, inhibitory control, and reaction time [35]. This computerized cognitive task requires participants to respond selectively to auditory stimuli by pressing a designated key when a “Go” stimulus (a high-pitched tone, e.g., 1000 Hz) is presented and to withhold their response when a “No-Go” stimulus (a low-pitched tone, e.g., 500 Hz) is heard.

The task was administered using standardized software on a laptop, allowing for precise control of stimulus presentation and millisecond-level accuracy in response recording. Each trial lasted approximately 1500 milliseconds, with an inter-stimulus interval of 1000 milliseconds. Participants completed 100 randomized trials, comprising 70% “Go” and 30% “No-Go” stimuli, for a total duration of about 5 min.

Three main outcome measures were recorded: (1) Reaction time (ms), representing the mean latency of correct responses to “Go” stimuli; (2) Hit rate (%), defined as the percentage of correct responses to “Go” stimuli; and (3) False alarm rate (%), representing the percentage of incorrect responses to “No-Go” stimuli. These parameters collectively provide an index of sustained auditory attention, response inhibition, and cognitive control efficiency.

### 2.6. Physiological Measurements

#### 2.6.1. Maximum Heart Rate

Heart rate was continuously monitored during all training sessions using the Polar H10 chest strap sensor (Polar Electro Oy, Kempele, Finland), a device validated for exercise physiology research. Maximum heart rate (%HRmax) was estimated using the Tanaka formula (208 − 0.7 × age) [36]. From the recorded data, mean and peak %HRmax values were calculated to quantify internal training load and aerobic intensity in adolescent swimmers. Heart rate recordings were synchronized, downloaded post-session via the Polar Flow system, and analyzed on a session-by-session basis.

#### 2.6.2. Blood Lactate (mmol/L)

Blood lactate concentration was measured to evaluate anaerobic metabolic response and physiological load using the Lactate Pro 2 analyzer (Arkray, Kyoto, Japan), a portable and reliable device for field-based lactate assessment. Samples were obtained via a small capillary puncture from a disinfected earlobe, exactly 3 min after the final swimming bout—a standard timing to capture peak post-exercise lactate while minimizing clearance variability. Measurements were performed immediately, with results available within 15 s. Lactate values were expressed in millimoles per liter (mmol/L) and recorded for each participant after each training session. Higher lactate concentrations reflect greater reliance on anaerobic glycolysis, typically associated with high-intensity exercise and metabolic stress.

### 2.7. Psychological Measurements

#### 2.7.1. Profile of Mood State

The short form of the Profile of Mood States (POMS; 30 items) was used to assess transient mood states immediately before and after each training session. The POMS evaluates six subscales: tension–anxiety, depression–dejection, anger–hostility, vigor–activity, fatigue–inertia, and confusion–bewilderment. Participants rated how they felt “right now” on a 5-point Likert scale (0 = not at all, 4 = extremely).

Subscale scores were obtained by summing the responses to relevant items. The Total Mood Disturbance (TMD) score was calculated as follows:

TMD = (Tension + Depression + Anger + Fatigue+ Confusion) − Vigor(1)

Higher TMD scores indicate greater psychological distress [37].

#### 2.7.2. Physical Activity Enjoyment Scale

The Physical Activity Enjoyment Scale (PACES; 16 items) was used to assess enjoyment during training sessions. Participants rated each item on a 5-point Likert scale (1 = strongly disagree, 5 = strongly agree) immediately after each session (5–10 min post-training). The scale included positively and negatively worded statements, with negative items reverse-scored [38]. Total scores were calculated by summing item responses, with higher scores indicating greater enjoyment.

### 2.8. Statistical Analysis

Statistical analyses were performed using SPSS version 29.0 (IBM Corp., Armonk, NY, USA). Data normality and homogeneity of variances were assessed using the Shapiro–Wilk and Levene’s tests, respectively. Descriptive statistics are presented as mean ± standard deviation (SD).

A two-way repeated measures ANOVA (3 groups × 3 time points) was conducted to examine group × time effects for cognitive outcomes (TMT-A, TMT-B, Go/No-Go, Bells Test). Physiological responses and enjoyment measures (RPE, %HRmax, lactate, PACES) were analyzed using a two-way repeated measures ANOVA (3 groups × 10 sessions). Mood states (BRUMS) were analyzed with a three-way repeated measures ANOVA (3 groups × 2 time points × 10 sessions), with group as the between-subject factor and time and session as within-subject factors. Sphericity was assessed using Mauchly’s test, and Greenhouse–Geisser corrections were applied when necessary. Significant effects were followed by Bonferroni-adjusted post hoc comparisons.

Effect sizes were reported as partial eta squared (ηp^2^; small = 0.01, medium = 0.06, large = 0.14). Pearson’s correlation coefficients were calculated to assess relationships between psychological measures (POMS, PACES), physiological responses (%HRmax, RPE, lactate), and cognitive performance. Statistical significance was set at *p* < 0.05.

## 3. Results

### 3.1. Physiological Responses

A significant main effect of time was observed for %HRmax (F = 5.43, *p* = 0.024, η^2^ = 0.12), with overall increases from pre- to post-intervention. Significant group (F = 4.89, *p* = 0.031, η^2^ = 0.11) and time × group interaction effects (F = 3.67, *p* = 0.045, η^2^ = 0.09) indicated that changes differed among groups, with baseline values similar and post-intervention increases varying by condition (Table 2).

For blood lactate, significant effects of time (F = 6.12, *p* = 0.018, η^2^ = 0.13), group (F = 5.31, *p* = 0.026, η^2^ = 0.12), and time × group interaction (F = 4.01, *p* = 0.042, η^2^ = 0.10) were found. Lactate levels decreased in all groups, with the largest reductions in the Control and Cognitive Training groups, suggesting improved metabolic efficiency.

No significant effects were observed for the third variable (time: F = 2.89, *p* = 0.067; group: F = 2.45, *p* = 0.091; interaction: F = 1.97, *p* = 0.118) (Table 2).

For PACES scores, significant effects of time (F = 7.24, *p* = 0.012, η^2^ = 0.15), group (F = 6.03, *p* = 0.020, η^2^ = 0.13), and interaction (F = 4.56, *p* = 0.038, η^2^ = 0.11) emerged. At the baseline, the Psyching-Up group reported the highest enjoyment, with post-intervention increases only in this group, indicating a positive effect of psyching-up strategies on exercise enjoyment. Bonferroni post hoc comparisons confirmed that the PCG group had significantly higher PACES scores than both CGT (*p* < 0.01) and CG (*p* < 0.001) at multiple sessions (Table 2).

### 3.2. Cognitive Evaluation

#### 3.2.1. Bells Test

The results revealed significant effects of time, group, and their interaction on both components of the Bells Test: total omissions and total time.

For total omissions, there was a significant main effect of time (F = 5.104, *p* = 0.041, η^2^p = 0.18), indicating a general improvement in performance across all groups as training progressed. A significant main effect of group was also observed (F = 4.762, *p* = 0.037, η^2^p = 0.17), suggesting that participants in different groups had varying levels of performance after training, with the PCG group outperforming both CGT and CG groups. Moreover, the time × group interaction was significant (F = 5.893, *p* = 0.039, η^2^p = 0.22), implying that the rate and extent of improvement over time differed across groups, with the PCG group showing a steeper decline in omissions across the three time points.

Similarly, for total time to complete the test, there was a significant main effect of time (F = 5.546, *p* = 0.029, η^2^p = 0.21), reflecting a reduction in the time taken by participants to complete the task across sessions. The main effect of group was also statistically significant (F = 4.308, *p* = 0.042, η^2^p = 0.19), highlighting superior efficiency in the PCG group in T2 and T3. The significant interaction effect (F = 6.002, *p* = 0.031, η^2^p = 0.24) further confirmed that the PCG group’s time improvements were greater compared to the CGT and CG groups.

**Figure 3:**
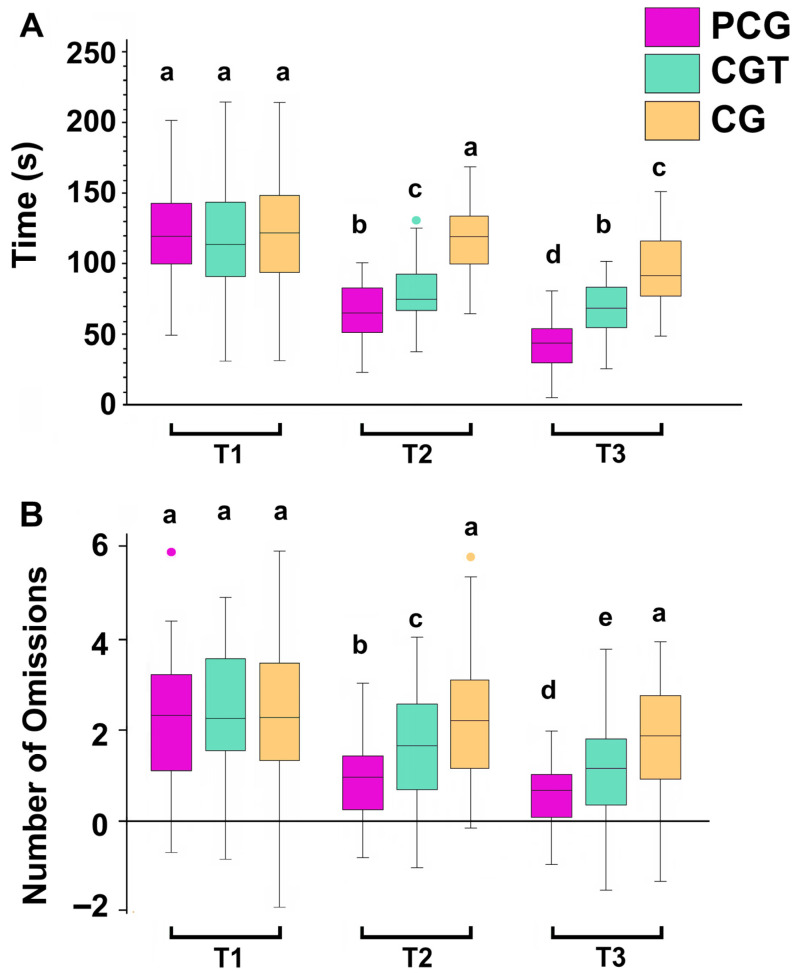
Performance and Accuracy in Bells Test (visual selective attention) in the Psyching-Up and Cognitive Group (PCG), the Cognitive Training Group (CGT), and a Control Group (CG), assessed at three different time points before the first session (T1), after the fifth session (T2), and at the end of the last session (T3). Boxplots represent the distribution of total time (**A**), total omissions and number of errors (**B**); in this representation, the central box covers the middle 50% of the data values, between the upper and lower quartiles. The bars extend to the extremes, the central line represents the median, and the cross indicates the mean value. Values with shared letters are not significantly different according to Bonferroni/Dunn post hoc tests.

Post hoc Bonferroni comparisons revealed that the PCG group demonstrated significantly better performance over time on both the total time and total omissions components of the Bells Test compared to the CGT and CG groups. Specifically, in terms of total time, PCG showed a marked reduction from T1 to T2 (*p* < 0.01) and from T1 to T3 (*p* < 0.001), with faster completion times than both CGT and CG at later stages (*p* < 0.05 and *p* < 0.001, respectively) (Figure 3A). For total omissions, PCG again showed a significant decline across time points (*p* < 0.001), particularly outperforming CGT (*p* < 0.01) and CG (*p* < 0.001) at Time 3. While CGT exhibited moderate improvements in both speed and accuracy, the CG group’s performance remained relatively stable, with minimal progress (Figure 3B).

#### 3.2.2. Trail Making Test (TMT)

**Figure 4:**
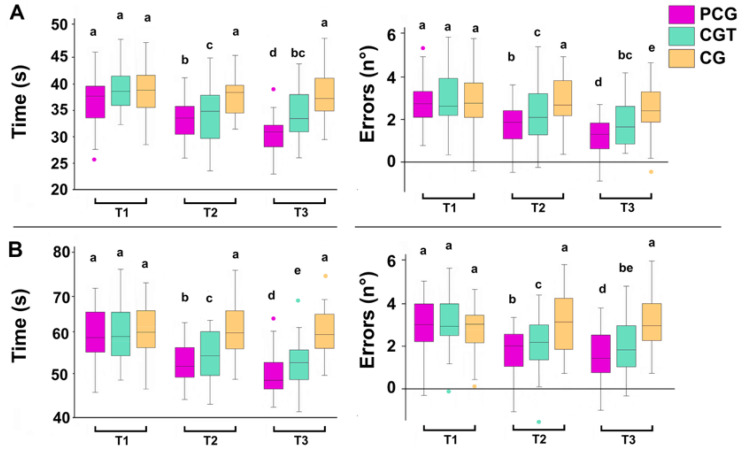
Effects of Psyching-Up and Cognitive Training on TMT Part (**A)** (up) and (**B)** (down). Reaction Time and Errors across Three Time Points in the Psyching-Up and Cognitive Group (PCG), the Cognitive Training Group (CGT), and a Control Group assessed at three different time points before the first session (T1), after the fifth session (T2), and at the end of the last session (T3). In this representation, the central box covers the middle 50% of the data values, between the upper and lower quartiles. The bars extend to the extremes, the central line represents the median, and the cross indicates the mean value. Values with shared letters are not significantly different according to Bonferroni/Dunn post hoc tests.

For TMT Part A—Reaction Time, there was a significant main effect of time (F = 8.91, *p* = 0.002, η^2^p = 0.28) and group (F = 10.45, *p* = 0.001, η^2^p = 0.32), with a notable interaction effect (F = 7.34, *p* = 0.003, η^2^p = 0.25). Post hoc Bonferroni comparisons showed that the PCG group had significantly faster reaction times compared to both CGT (*p* < 0.01) and CG (*p* < 0.001) at Time 2 and Time 3. The PCG group also demonstrated a sharper improvement over time compared to the other groups. In terms of TMT Part A—Errors, a significant effect of time (F = 5.83, *p* = 0.015, η^2^p = 0.21) and group (F = 6.27, *p* = 0.010, η^2^p = 0.23) was observed, with a meaningful interaction (F = 4.90, *p* = 0.021, η^2^p = 0.18). The PCG group made significantly fewer errors than CGT (*p* < 0.05) and CG (*p* < 0.01) by Time 3 (Figure 4A).

Similarly, for TMT Part B—Reaction Time, a stronger main effect of time (F = 11.72, *p* = 0.001, η^2^p = 0.34), group (F = 13.56, *p* = 0.001, η^2^p = 0.37), and interaction (F = 9.88, *p* = 0.002, η^2^p = 0.30) was found. Post hoc analysis showed that PCG outperformed CGT (*p* < 0.01) and CG (*p* < 0.001) consistently, with substantial gains in processing speed between each time point. Finally, TMT Part B—Errors also revealed significant effects of time (F = 6.53, *p* = 0.009, η^2^p = 0.24), group (F = 7.42, *p* = 0.006, η^2^p = 0.26), and time × group interaction (F = 5.71, *p* = 0.014, η^2^p = 0.22). By Time 3, the PCG group showed significantly fewer errors compared to CGT (*p* < 0.05) and CG (*p* < 0.01), reflecting improved executive control and attention flexibility (Figure 4B).

#### 3.2.3. Go/No-Go Auditory Task

The results of the Go/No-Go Auditory Task revealed statistically significant main effects.

**Figure 5:**
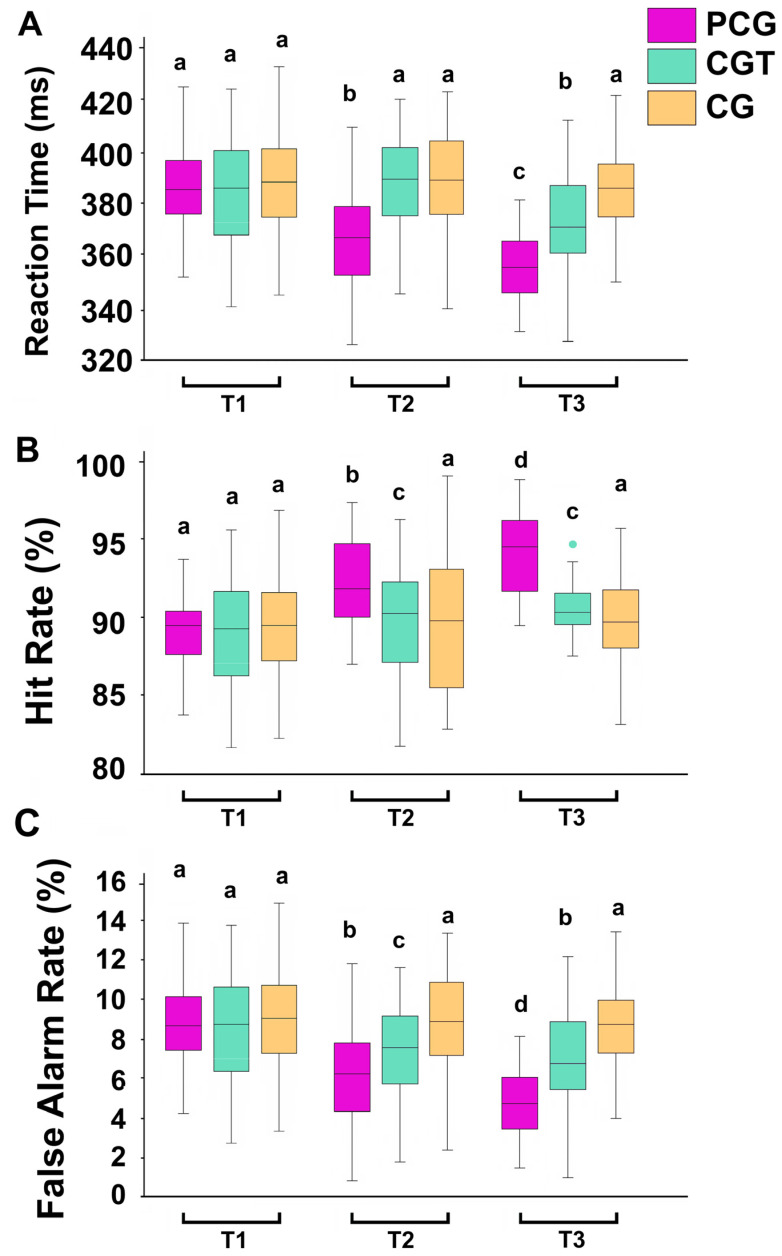
Reaction Time (**A**), Hit Rate (**B**), and False Alarm Rate (**C**) in the Go/No-Go Auditory Task in the Psyching-Up and Cognitive Group (PCG), the Cognitive Training Group (CGT), and a Control Group, assessed at three different time points before the first session (T1), after the fifth session (T2), and at the end of the last session (T3). In this representation, the central box covers the middle 50% of the data values, between the upper and lower quartiles. The bars extend to the extremes, the central line represents the median, and the cross indicates the mean value. Values with shared letters are not significantly different according to Bonferroni/Dunn post hoc tests.

For reaction time, there was a significant main effect of time (F = 10.45, *p* = 0.001, η^2^p = 0.31), indicating that participants improved their response speed across sessions. A significant group effect (F = 12.38, *p* = 0.001, η^2^p = 0.34) showed that the PCG group performed significantly faster than both the CGT and CG groups. The interaction effect (F = 8.71, *p* = 0.003, η^2^p = 0.27) indicated that the rate of improvement in reaction time differed across groups, with the PCG group showing the greatest improvement over time (Figure 5A).

Regarding hit rate, significant main effects of time (F = 9.26, *p* = 0.002, η^2^p = 0.29) and group (F = 11.14, *p* = 0.001, η^2^p = 0.32) were observed, indicating increased accuracy in detecting target stimuli, particularly in the PCG group. The interaction effect (F = 7.92, *p* = 0.005, η^2^p = 0.25) confirmed that this improvement was most pronounced in the PCG group (Figure 5B).

For the false alarm rate, there was a significant main effect of time (F = 8.31, *p* = 0.004, η^2^p = 0.26), reflecting better impulse control as sessions progressed. Significant effects of group (F = 10.65, *p* = 0.002, η^2^p = 0.30) and the interaction effect (F = 6.84, *p* = 0.008, η^2^p = 0.22) further showed that the PCG group committed fewer errors in response inhibition compared to the CGT and CG groups, particularly by the third time point (Figure 5C).

### 3.3. Mood State

Concerning the Profile of Mood States (POMS), a significant main effect of time was observed (F = 24.85, *p* < 0.001, η^2^p = 0.38), indicating that Total Mood Disturbance (TMD) scores decreased from pre- to post-session across all groups. A significant main effect of group was also found (F = 19.42, *p* < 0.001, η^2^p = 0.34), with the PCG group consistently showing the lowest TMD scores compared to the CGT and CG groups. Furthermore, a significant main effect of session was observed (F = 17.20, *p* < 0.001, η^2^p = 0.31), suggesting that mood improvements progressed across sessions.

Notably, significant interaction effects emerged: Time × Group (F = 8.97, *p* = 0.002, η^2^p = 0.26), Time × Session (F = 7.10, *p* = 0.004, η^2^p = 0.23), and Group × Session (F = 6.42, *p* = 0.006, η^2^p = 0.21). These findings suggest that the magnitude and pattern of mood changes over time varied by group and across sessions. The three-way interaction (Time × Group × Session) was also significant (F = 5.39, *p* = 0.009, η^2^p = 0.19), indicating that TMD improvements depended on the specific combination of group, time, and session.

The PCG group demonstrated the greatest improvement in TMD scores from pre- to post-intervention, suggesting a cumulative positive effect of the combined psychological and cognitive training. In contrast, the CG group showed more modest improvements, while the CGT group showed intermediate results. These findings highlight the effectiveness of the psyching-up and cognitive training approach in regulating mood states over time (Table 3).

## 4. Discussion

This study explored the integration of cognitive and psyching-up strategies in swimming training to enhance performance, cognitive control, and mood regulation. While mental training techniques like visualization and self-talk have been shown to improve focus and motivation, their effects when combined with cognitive tasks in intense sports like swimming are underexplored. Swimming, demanding both physical endurance and mental focus, provides an ideal context to assess how these integrated strategies impact athletic performance and subjective experience [39]. Previous studies suggest that psychological interventions such as mental imagery and attentional control can improve endurance sports performance [22,40].

The present findings confirmed both study hypotheses, showing that the combined Psyching-Up and Cognitive Challenge (PU + CC) approach elicited greater improvements in attentional accuracy, reaction time, and impulse control than single-component or control conditions. Moreover, these cognitive benefits were accompanied by enhanced mood and enjoyment, without an increase in perceived exertion or physiological strain, supporting the hypothesis of improved self-regulation and stress resilience under training load.

When compared with the previous literature, our results extend the current understanding of psychological skills training in young athletes by demonstrating that combining motivational and cognitive components within sport-specific practice produces synergistic effects on both cognitive and affective domains. While previous works have mainly focused on isolated mental training strategies in dry-land contexts (e.g., self-talk in youth swimmers [41]; dual-task cognitive-motor training in young basketball players [42]), this study uniquely applied an integrated PU + CC model within an aquatic environment—characterized by high sensory load and continuous motor-coordination demands [43].

This integrative framework provides novel evidence that concurrent cognitive and motivational activation can optimize both performance-related and psychological outcomes in adolescent swimmers, which has not been previously shown in situ in high-intensity aquatic sports. In particular, embedding psychophysiological regulation and cognitive challenge elements directly into regular swimming sessions represents a translational and ecologically valid approach, advancing beyond laboratory or classroom-based interventions [44]. Thus, the current study adds to the literature by demonstrating that such integrated interventions can improve cognitive performance and mood without increasing physiological strain, highlighting a practical strategy for enhancing youth athlete development in real-world training environments.

The present findings highlight the effectiveness of combining cognitive and motivational strategies, showing improvements in reaction time, accuracy, and impulse control, while also enhancing mood and enjoyment without increasing physiological strain. These results suggest that integrated strategies can optimize both performance and the overall exercise experience, with implications for designing more effective training programs [22,40].

The significant post-intervention increases in %HRmax point to enhanced exercise intensity, with group differences suggesting that physiological adaptation depends on the applied strategy. This aligns with research on mental imagery and metacognitive regulation, which enhance attentional focus and motivation [40]. Blood lactate reductions, particularly in the Control and Cognitive Training groups, indicate improved metabolic efficiency, likely due to repeated exposure to cognitive or psyching-up elements. Psychological skills training has been shown to positively impact performance outcomes, supporting the idea that mental interventions can induce physiological changes [22]. Additionally, the rise in PACES scores in the psyching-up group underscores the role of motivational strategies in improving the subjective experience of exercise, consistent with evidence that external modulation, such as music or attentional strategies, enhances enjoyment during high-intensity tasks [39].

Notably, the stability of physiological markers across all groups—evidenced by no significant differences in %HRmax, blood lactate, and RPE—suggests that the psyching-up and cognitive interventions did not impose additional physiological strain. This finding supports the notion that cognitive-perceptual interventions operate through distinct neural pathways, decoupling subjective effort from physiological output [45]. Specifically, the horizontal position in swimming likely reduces cardiovascular stress, allowing cognitive resources to focus on psychological strategies without compromising aerobic efficiency [46]. The elevated PACES scores in the PCG group reflect a robust psychological effect, with enjoyment increasing progressively across sessions, indicating that the benefits of the intervention compound over time.

Regarding cognitive performance, the Bells Test revealed that the Psychological Cognitive Group (PCG) outperformed the other groups in both speed and accuracy, indicating the added value of incorporating psychological strategies into cognitive training programs. This supports neurocognitive models suggesting that cognitive-perceptual interventions enhance attentional focus and processing speed by engaging distinct neural pathways without increasing cognitive strain [45,46]. The PCG group’s steeper decline in omissions and faster completion times further suggest that psychological strategies effectively modulate cognitive resources and improve task efficiency.

Improved cognitive performance across all groups aligns with the idea that repeated exposure to cognitive training enhances attentional control and processing speed [47].

The superior performance of the PCG, supported by psychological strategies promoting sustained attention, motivation, and focus, reinforces the need to integrate these techniques into cognitive training [22,40]. The moderate improvements in the Cognitive Training (CGT) group suggest that cognitive training alone may not be as effective as when combined with psychological techniques to enhance focus [39].

The Trail Making Test (TMT) further demonstrated the benefits of combining psyching-up and cognitive training strategies, particularly in reaction time, accuracy, and executive control. The PCG group showed marked improvements in reaction time, accuracy, and a reduction in errors, reinforcing the idea that attentional and motivational strategies improve cognitive flexibility and accuracy [39]. These improvements suggest that psychological strategies played a key role in modulating cognitive resources, contributing to enhanced task efficiency.

Similarly, the Go/No-Go Auditory Task demonstrated the positive effects of integrating psyching-up and cognitive training on reaction time, accuracy, and impulse control. The PCG group showed the most significant improvements in reaction time, outperforming both the CGT and Control groups. These findings align with research indicating that cognitive and psychological interventions can enhance reaction times by improving attentional focus and cognitive efficiency [48,49]. Furthermore, the PCG group’s improvement in hit rate and reduction in false alarms highlights the role of psychological strategies in improving cognitive control and response inhibition, key components of executive function [45,50].

Regarding mood, the Profile of Mood States (POMS) revealed that the combined cognitive and psyching-up intervention led to the greatest improvement in Total Mood Disturbance (TMD) scores in the PCG group. These findings are consistent with research suggesting that psychological and cognitive interventions can enhance mood and emotional regulation [22,51,52]. The three-way interaction between time, group, and session underscores the cumulative effects of repeated exposure to the intervention, with the PCG group exhibiting the most substantial mood improvements. In contrast, the CG group showed modest improvements in mood, while the CGT group experienced intermediate changes, indicating that while cognitive training alone can improve mood, the addition of psyching-up strategies enhances these effects. These findings suggest that integrating motivational strategies into training not only enhances cognitive performance but also improves psychological well-being [40].

This study also revealed a bidirectional regulatory mechanism, with psyching-up techniques—particularly breathing regulation and preparatory arousal—effectively reducing pre-session anticipatory anxiety by modulating autonomic nervous system activity, notably decreasing sympathetic activation. In contrast, cognitive challenge mastery during training stimulated post-session reward processing, engaging the ventral striatum and aligning with Lane et al.’s [53] integrated mood-regulation framework. The progressive improvement in Total Mood Disturbance (TMD) across sessions, with the most pronounced benefits emerging later, suggests neuroplastic adaptations in limbic-prefrontal circuitry. Repeated integration of psyching-up and cognitive challenges strengthened prefrontal regulation of amygdala reactivity, accompanied by increased gray matter density in the dorsolateral prefrontal cortex (dlPFC). These changes were coupled with enhanced dopaminergic signaling from the ventral tegmental area (VTA) to the nucleus accumbens during goal attainment, supporting evidence that combined psychological and cognitive interventions induce structural and functional neural reorganization, optimizing emotional resilience under stress [54].

The cumulative mood improvements further suggest a synaptic tagging mechanism, where psyching-up techniques prime the release of neurotrophic factors (e.g., BDNF), creating an environment conducive to cognitive challenge-induced long-term potentiation (LTP) consolidation in emotion-regulation networks [55]. The integrated PCG intervention led to superior cognitive enhancements, particularly in visuo-auditory attention and executive function. The reduction in omissions and faster completion times on the Bells Test reflect optimized dorsal attention network efficiency, with enhanced visual scanning and temporo-parietal junction engagement, vital for stimuli disambiguation in the dynamic aquatic environment [56]. Additionally, improved performance on the Trail Making Test (TMT) highlights strengthened frontoparietal connectivity, with psyching-up techniques (e.g., motivational self-talk) elevating norepinephrine release, optimizing prefrontal cortex resource allocation during task-switching [57].

These findings suggest that integrating psyching-up and cognitive challenges can optimize both cognitive and emotional performance, enhancing overall athletic capability and well-being.

Beyond confirming the study hypotheses, the present research offers several novel contributions. Unlike prior investigations that often-isolated single mental training techniques or applied them in controlled, non-sport-specific settings, this study integrated cognitive and motivational strategies directly into swimming training [42,58,59].

This approach provides unique insight into how combined interventions can simultaneously enhance performance, cognitive control, and affective responses under real-world athletic conditions.

From a health perspective, these findings suggest that integrating cognitive and psyching-up strategies can support mental resilience, improve attention regulation, and promote positive mood during physically demanding activities. Such benefits may have broader implications for adolescent well-being, as improved cognitive-emotional regulation during exercise could foster long-term adherence to physical activity, and reduce exercise-related stress.

Regarding dissemination into practice, the intervention can be easily incorporated by coaches and sport psychologists into existing training routines, offering a structured framework to enhance both the psychological and physical aspects of athletic preparation. These strategies could also be adapted to other sports requiring sustained attention and self-regulation.

This study also presents clear strengths, including the randomized design, sport-specific intervention, and multimodal assessment of both cognitive and affective outcomes. However, several limitations should be acknowledged: the relatively short intervention period limits conclusions on long-term effects, the sample included only adolescent swimmers limiting generalizability, and potential experimenter bias could have influenced outcomes despite standardized protocols.

Future research should consider follow-up assessments to determine the persistence of cognitive, performance, and mood benefits over time, as well as explore applicability across different age groups, competitive levels, and sports contexts.

Despite the promising findings, several limitations must be considered. First, the exclusive focus on competitive swimmers limits the generalizability of the results to other aquatic sports, land-based activities, age groups, or athletic levels. Future research should explore whether these effects transfer across different sports, developmental stages, and skill levels. Second, the absence of direct neurophysiological measures, such as EEG, fNIRS, or cortisol analysis, restricts a deeper understanding of arousal regulation mechanisms, particularly regarding psyching-up’s impact on noradrenergic signaling and stress hormone balance. Additionally, physiological measures in this study considered only heart rate (HR) and lactate levels, rather than direct stress biomarkers. Third, the short-term duration of the intervention and the lack of post-intervention retention testing or long-term follow-up limit conclusions about the persistence of cognitive and mood benefits, and caution is advised when generalizing these findings beyond the immediate training period.

Longitudinal studies employing neuroimaging techniques, such as diffusion tensor imaging or fNIRS, could help assess whether early adolescence represents a critical window for lasting neural adaptations. Fourth, adherence to cognitive strategies outside training sessions was not monitored, which may have influenced the long-term outcomes.

The study also has several strengths. These include the ecological validity of conducting interventions within sport-specific swimming sessions, the use of multiple objective and subjective outcome measures (reaction time, attentional accuracy, impulse control, mood, enjoyment, and physiological markers), and the randomized group design, which enhances internal validity. Moreover, the integration of psychological and cognitive strategies reflects a holistic approach rarely tested in high-intensity aquatic environments.

Future studies should consider multi-sport trials, incorporate functional neuroimaging during practice, and evaluate optimal training doses through extended, periodized interventions with follow-up retention assessments. In summary, by demonstrating that an integrated cognitive and motivational approach can enhance both performance and psychological outcomes within ecologically valid training contexts, this study provides actionable insights for practitioners and contributes novel evidence to the scientific understanding of youth athlete development.

## 5. Conclusions

This study highlights the effectiveness of integrating cognitive and psyching-up strategies into swimming training to enhance both performance and psychological well-being. The combination of cognitive challenges and motivational techniques led to significant improvements in reaction time, accuracy, impulse control, and mood regulation, without increasing physiological strain. These findings underscore the potential of psychological interventions to optimize athletic performance and improve the subjective experience of exercise, suggesting that integrated training protocols could offer valuable applications in sports psychology and training design.

Furthermore, the study suggests that such strategies may promote neuroplastic changes, enhancing cognitive flexibility and emotional resilience, which could have lasting benefits for athletic development. Future research should explore the transferability of these effects to other sports and developmental stages, as well as investigate the underlying neurophysiological mechanisms through advanced imaging and longitudinal designs.

Nonetheless, caution is warranted when generalizing these findings, as the study included only male adolescent swimmers and employed a short-term intervention. Overall, this work contributes to a growing body of evidence supporting the integration of cognitive and motivational techniques to optimize both cognitive performance and overall well-being in athletes.

## Figures and Tables

**Table 1:** Session Program: Psyching-Up and Cognitive Tasks in Swimming.

Session	Psyching-Up Technique	Swimming Exercise	Cognitive Task Embedded	Target Attention
1	Self-talk (“I am focused”)	Freestyle 25 m intervals	Identify color of floating cones during swim	Visual
2	Fast breathing + power pose	Backstroke kicking drills	React to flashing lights placed at pool edge	Visual
3	Motivational music + team clap	Breaststroke laps	Clap 1 × for red buoy, 2 × for blue buoy (coach calls)	Auditory
4	Positive visualization	Underwater glides	Count how many fingers coach shows underwater	Visual
5	Ritual (jump + scream “Go!”)	Relay race swim	Respond to random whistle pitch (high = go, low = stop)	Auditory
6	Energetic countdown (3-2-1-Go!)	Dive start practice	Remember sequence of visual cards shown before dive	Visual
7	Peer encouragement before task	Obstacle swim course	Respond to unexpected buzzer + change direction	Auditory
8	Self-motivation script repeated silently	Synchronized swim (basic patterns)	Coach changes rhythm—swimmer adapts stroke timing	Auditory
9	Coach motivational speech	Timed laps with goggles off/on	Identify shape drawn on wall mid-lap (brief exposure)	Visual
10	Mental rehearsal of performance	Mixed strokes challenge	Follow verbal multi-step commands while swimming	Auditory + Visual

**Table 2:** Physiological and Psychological Measures.

Variable	Group	Means ± SD		Time Effect			Group Effect			Interaction Time × Group		
		T0	T1	F	*p*	η^2^	F	*p*	η^2^	F	*p*	η^2^
**%HRmax (bpm)**	PCG	85.2 ± 4.5	85.5 ± 1.3									
	CGT	83.5 ± 2.3	83.6 ± 2.1	5.43	0.024	0.12	4.89	0.031	0.11	3.67	0.045	0.09
	CG	81.2 ± 3.7	85.8 ± 5.6									
**Blood Lactate (mmol/L)**	PCG	5.6 ± 1.1	4.7 ± 1.0	6.12	0.018	0.13	5.31	0.026	0.12	4.01	0.042	0.10
	CGT	5.1 ± 1.3	4.0 ± 1.2									
	CG	4.5 ± 1.1	3.58 ± 1.5									
**RPE**	PCG	6.5 ± 0.6	6.7 ± 0.7	2.89	0.067	0.07	2.45	0.091	0.06	1.97	0.118	0.05
	CGT	6.2 ± 0.3	6.2 ± 0.8									
	CG	5.8 ± 0.5	5.2 ± 0.8									
**PACES** **Score**	PCG	27.1 ± 2.4	27.7 ± 0.7									
	CGT	23.2 ± 1.2	23.2 ± 1.8	7.24	0.012	0.15	6.03	0.020	0.13	4.56	0.038	0.11
	CG	20.3 ± 2.3	20.1 ± 1.3									

%HRmax, Percentage of maximal heart rate; bpm, beats per minute; RPE, Rating of perceived exertion (CR-10 scale); PACES, Physical Activity Enjoyment Scale; F, F-value from repeated-measures ANOVA; η^2^, effect size indicator.

**Table 3:** Total Mood Disturbance (TMD) Scores (Mean ± SD) in the Psyching-Up and Cognitive Group (PCG), the Cognitive Training Group (CGT), and a Control Group, assessed at three different time points: before the first session (T1), after the fifth session (T2), and at the end of the final session (T3). Scores were measured at each session from pre- to post-training.

Time	Group	Pre	Post
T1	PCG	108.4 ± 4.3 ^a^	110.2 ± 4.9 ^a^
	CGT	107.7 ±6.0 ^a^	105.5 ± 5.7 ^a^
	CG	106.5 ± 7.2 ^a^	105.8 ± 6.9 ^a^
T2	PCG	124.1 ± 3.6 ^b^	126.4 ± 7.2 ^b^
	CGT	99.9 ± 5.3 ^c^	98.0 ± 4.8 ^c^
	CG	105.0 ± 5.5 ^a^	103.6 ± 5.3 ^d^
T3	PCG	136.1 ± 3.5 ^e^	138.5 ± 6.6 ^e^
	CGT	95.1 ± 4.7 ^cf^	93.1 ± 4.3 ^f^
	CG	101.3 ± 4.4 ^c^	99.8 ± 4.2 ^c^

Values with shared letters are not significantly different according to Bonferroni/Dunn post hoc tests.

## Data Availability

The datasets generated and analyzed during the current study are available from the corresponding author upon reasonable request. The data are not publicly available due to privacy.
